# Patterns and Predictors of Relapse Following Radical Chemoradiation Therapy Delivered Using Intensity Modulated Radiation Therapy With a Simultaneous Integrated Boost in Anal Squamous Cell Carcinoma

**DOI:** 10.1016/j.ijrobp.2019.10.016

**Published:** 2020-02-01

**Authors:** Rebecca Shakir, Richard Adams, Rachel Cooper, Amy Downing, Ian Geh, Duncan Gilbert, Clare Jacobs, Christopher Jones, Cressida Lorimer, Wanangwa C. Namelo, David Sebag-Montefiore, Paul Shaw, Rebecca Muirhead

**Affiliations:** ∗ Oxford Cancer and Haematology Centre, Oxford University Hospitals, Oxford, United Kingdom; † Nuffield Department of Population Health, University of Oxford, Oxford, United Kingdom; ‡ Velindre Cancer Centre, Cardiff, United Kingdom; § Radiotherapy Research Group, Leeds Cancer Centre, St James’s University Hospital, Leeds, United Kingdom; ‖ Leeds Institute for Medical Research at St James, University of Leeds, United Kingdom; # Queen Elizabeth Hospital, University Hospitals Birmingham NHS Foundation Trust, Birmingham, United Kingdom; ∗∗ Sussex Cancer Centre, Royal Sussex County Hospital, Brighton, United Kingdom; †† School of Molecular & Cellular Biology, Faculty of Biological Sciences, University of Leeds, Leeds, United Kingdom; ‡‡ Leeds Clinical Trials Unit, Faculty of Medicine & Health, University of Leeds, United Kingdom; §§ School of Biosciences, Cardiff University, United Kingdom

## Abstract

**Purpose:**

Our purpose was to describe the patterns and predictors of treatment failure in patients receiving definitive chemoradiation therapy (CRT) for anal squamous cell carcinoma (ASCC), delivered using intensity modulated radiation therapy (IMRT).

**Methods and Materials:**

Our study was a retrospective cohort analysis of consecutive patients treated with curative intent for ASCC using CRT delivered with a standardized IMRT technique in 5 UK cancer centers. Patients were included from the start of UK IMRT guidance from February 2013 to October 31, 2017. Collected data included baseline demographics, treatment details, tumor control, sites of relapse, and overall survival. Statistical analysis to calculate outcomes and predictive factors for outcome measures were performed using SPSS and R.

**Results:**

The medical records of 385 consecutive patients were analyzed. Median follow-up was 24.0 months. Within 6 months of completing CRT, 86.7% of patients achieved a complete response. Three-year disease-free survival and overall survival were 75.6% and 85.6%, respectively. Of all relapses, 83.4% occurred at the site of primary disease. There were 2 isolated relapses in regional nodes not involved at outset. Predictive factors for cancer recurrence included male sex, high N-stage, and failure to complete radiation therapy as planned.

**Conclusions:**

The treatment results compare favorably to published outcomes from similar cohorts using 3-dimensional conformal CRT. The observed patterns of failure support the current UK IMRT voluming guidelines and dose levels, highlighting our prophylactic nodal dose as sufficient to prevent isolated regional relapse in uninvolved nodes. Further investigation of strategies to optimize CR should remain a priority in ASCC because the site of primary disease remains the overwhelming site of relapse.

**Summary**

To inform intensity modulated radiation therapy delivery and future trials in anal squamous cell carcinoma, we performed a retrospective analysis of a UK population of 385 patients, treated with national guidance, and report patterns and predictors of failure. Our guidance results in a negligible regional relapse rate in areas with no disease at outset, with a high relapse at the primary site. Our study supports efforts to maximize local disease management in the post intensity modulated radiation therapy era.

## Introduction

Anal squamous cell carcinoma (ASCC) is an uncommon malignancy, with an annual incidence of approximately 8500 in the United States and 1500 in the United Kingdom.^1^, ^2^, ^3^, ^4^ Chemoradiation therapy (CRT) with concurrent mitomycin-c and 5-fluorouracil has been established as the standard of care after 6 large international trials.^5^, ^6^, ^7^, ^8^, ^9^, ^10^, ^11^, ^12^

In the last decade, there has been increasing use of intensity modulated radiation therapy (IMRT).^13^, ^14^, ^15^, ^16^, ^17^ Various protocols have been developed internationally, with differences in doses, volumes, and constraints.^18^, ^19^, ^20^, ^21^ The UK IMRT guidance was derived from the principles of the UK ACT2 phase 3 trial.^21^ UK IMRT demonstrated improved toxicity and overall treatment time compliance compared with 3-dimensional (3D) conformal radiation therapy (RT) treatment in a large national audit.^22^ There have been some small iterations; however, the guidance remains very similar to that at conception in February 2013.

The transition from 3D conformal RT to IMRT presents a number of challenges. Radiation Therapy Oncology Group (RTOG) 0529, a single-arm phase 2 trial investigating the toxicity of IMRT in ASCC, included pretreatment quality assurance on all RT treatment plans and reported a replanning rate of 81%.^14^ In addition, the lack of data on relapse patterns and the difference in current atlases highlight the potential for further data to inform and improve delineation guidelines. Lastly, the prophylactic nodal dose was previously delivered in doses of 1.8 to 2 Gy/fraction for the first phase of the CRT. With the development of IMRT protocols, the prophylactic dose is delivered throughout RT in smaller doses per fraction. The optimal prophylactic dose is unknown.

The goal of this study was to determine the patterns and predictors of failure after IMRT delivery in ASCC, in addition to quantifying the core outcome measures for ASCC in this group, and to inform routine practice and guidelines while potentially highlighting areas of interest for future research and development.

## Methods and Materials

### Patients

Medical records were retrospectively reviewed for patients with anal canal and anal margin squamous cell carcinoma who underwent definitive RT using an IMRT technique from February 2013 to February 2018 in 5 UK Cancer Centres: Oxford Cancer and Haematology Centre, Leeds Cancer Centre, Queen Elizabeth Hospital, Birmingham; Sussex Cancer Centre, Brighton and Velindre Cancer Centre, Cardiff. Routine diagnostic imaging over this timeframe was magnetic resonance imaging (MRI) of the pelvis and computed tomography (CT) of the chest, abdomen, and pelvis. The use of positron emission tomography (PET) was heterogeneous, with 1 center using it for all ≥T2 patients, 3 using it in patients with staging discrepancies, and the last having no access. This study only included patients of sufficiently high risk to merit prophylactic nodal irradiation. Therefore, selected good-prognosis patients (T1 N0, well differentiated pathology, female patients, anal verge rather than canal) who underwent involved field radiation alone were excluded. Those with metastatic disease at diagnosis were excluded.

A standardized datasheet was developed and piloted by 3 centers. All centers used local electronic systems to identify all sequential ASCC undergoing RT during this timeframe and screened for eligibility. Data on patient, disease and treatment characteristics, and clinical outcomes including locoregional failure and survival were recorded. We present nodal staging according to the American Joint Commission on Cancer staging system TNM 7th and 8th editions.^23^^,^^24^

### Treatment

All treatment was delivered according to the UK IMRT guidance document.^25^ Full details are available at www.analimrtguidance.co.uk. In summary, all patients are treated supine; as such, the buttocks usually provide bolus to the anal verge, and in patients with disease infiltrating into the skin a Pyrex sheet is placed under the patient to provide build-up. All treatments were delivered in 28 fractions, with T1/2 N0 receiving 50.4 Gy to the gross primary tumor and T1/2 N+ or T3/4 Nany receiving 53.2 Gy.

Involved nodes received 50.4 Gy if <3 cm and 53.2 Gy if >3 cm; uninvolved pelvic nodes (CTV_Elective) including mesorectal, obturator, external and internal iliac, inguinal, and presacral received 40 Gy since March 2014 (previously 39.2 Gy). The ischiorectal fossa (IRF) was only included in the prophylactic dose level if the tumor involved the IRF on MRI. The superior margin of the CTV_Elective was 20 mm above the inferior aspect of the sacroiliac joint or 15 mm above the most superior site of gross tumor, whichever was most superior. Total margin from primary tumor gross tumor volume to planning target volume was 2.5 cm, reduced in 2016 in early node negative tumors to 2.0 cm. The clinical target volume (CTV) to planning target volume for the elective volume was 10 mm until April 2016 and 5 mm thereafter.

Treatment was delivered using inverse planned simultaneous integrated boost technique delivered with coplanar beams or arc delivery as per ICRU 83. An advanced convolution superposition algorithm was used. Suggested IMRT beam positions and optimal and mandatory constraints are detailed in the guidance.^25^ The guidance suggests mitomycin-c 12 mg/m^2^ day 1 with either capecitabine 825 mg/m^2^ twice dialy on days of RT or 5-fluorouracil 1000 mg/m^2^ on days 1 to 4 and 29 to 32. The verification protocol, which is detailed in the guidance, is daily online imaging with cone beam CT days 1 to 5 and weekly with orthogonal paired kV or MV images on all other days.

After completion of CRT, all patients underwent a physical examination at 3 months and regularly thereafter until complete response (CR) or a decision on salvage surgery was made. At least 1 MRI scan was performed to confirm radiologic CR at 3 months. Patients underwent clinical examinations every 3 months for 2 years, every 6 months for year 3, and in some centers yearly until 5 years. CT was typically done annually at the end of years 1, 2, and 3. Any concerns regarding symptoms or clinical examination would prompt further investigations. Anoscopy was not routinely performed as part of follow-up in any of the centers. Biopsy to confirm recurrence was performed when a salvage abdominoperineal resection (APR) was planned. Metastatic recurrence was diagnosed on radiologic evidence.

### Data processing

*CR* was defined as the absence of disease within the irradiated volume at 6 months based on clinical, radiologic, and pathologic examination. *Persistent disease* was failure to achieve CR.

*Locoregional recurrence* (LRR) includes all failures at site of primary tumor, within the pelvis or inguinal nodes, with or without distant failure, including both patients who failed to achieve CR at 6 months and those occurring more than 6 months after completion of CRT after initial CR. *Local failure* was defined as persistence or recurrence at the site of initial primary tumor and *regional failure* as persistence or recurrence elsewhere in the pelvis or inguinal nodes at any point. The site of failure was determined based on physical examination, imaging, and pathology. The relationship between RT volume and location of recurrence was determined by comparison between CT plans and diagnostic imaging.

*Distant relapse* was defined as development of disease outside of the pelvis or inguinal nodes independent of locoregional status at any point. Failure within the common iliac nodes was considered distant failure.

Disease-free survival was calculated in all patients, with an event defined as either a failure to achieve CR at 6 months or subsequent relapse (local, regional, or distant).

An interruption in RT treatment was defined as any extension to the treatment >2 days over the planned overall treatment time, as per RCR guidance.^26^

*Time to failure* was the interval from start of CRT to date of detection of recurrence. Last follow-up was considered the last clinic visit or date of death.

### Statistical analysis

Statistical analysis was carried out using IBM SPSS for Mac (version 25; SPSS Inc, Chicago, IL) and R (version 3.5.2; R Core Team, R Foundation for Statistical Computing, Vienna, Austria), and figures were produced using Microsoft Excel for Mac (version 15.33).

Three-year disease-free survival and overall survival were estimated by Kaplan-Meier methods. Cox proportional hazards regression analysis was performed to identify significant predictors of time to locoregional and distant recurrence, disease-free survival, and overall survival. Logistic regression analysis was performed to identify significant predictors of persistent disease at 6 months. The predictors used were age, sex, performance status, T stage, N stage (TNM 7 and 8), failure to complete RT as planned and type of chemotherapy. It was not possible to include HIV status in the analysis because almost 40% of patients in our cohort were not tested. All analyses were run as univariable tests and multivariable models. There was a small, time-dependent effect relating to RT completion, but it was not possible to include this as a time-varying covariate owing to the small number of patients failing to complete RT. For chemotherapy type, very few patients had single-agent treatments; these were therefore combined into a single category. For multivariable models, nodal status from TNM 7 was used in preference to TNM 8 because of the wider distribution of factor levels (only 1 patient had nodal status 1b). All patients were included in all models. For analyses where death was the endpoint, patients were censored at last known follow-up. For analyses of recurrence, patients were censored at last known follow-up or death.

A *P* value of <.05 was considered statistically significant.

### Governance approval

In accordance with UK practice for health care audits, approval for data collection was obtained locally by each contributing site’s Divisional Governance committee.

## Results

Three hundred eighty-five consecutive patients meeting the inclusion criteria were identified. Patient and tumor characteristics are shown in Table 1. All data fields were complete aside from performance status in 7, which was not documented at presentation.

**Table 1:** Patient and tumor characteristics (n = 385)

	n (% of total)
Sex	
Male	112 (29.1)
Female	273 (70.1)
Age, y	
Median	62
Range	29-88
HIV	
Positive	16 (4.2)
Negative	220 (57.1)
Not tested	149 (38.7)
Performance status	
0	212 (55.1)
1	141 (36.6)
2	19 (4.9)
3	6 (1.6)
Not documented	7 (1.8)
T stage	
Tx	1 (0.3)
T1	46 (11.9)
T2	174 (45.2)
T3	92 (23.9)
T4	72 (18.7)
N stage (TNM 7)	
Nx	1 (0.3)
N0	185 (48.1)
N1	72 (18.7)
N2	66 (17.1)
N3	61 (15.8)
N stage (TNM 8)	
Nx	1 (0.3)
N0	185 (48.1)
N1a	168 (43.6)
N1b	1 (0.3)
N1c	30 (7.8)
M stage	
Mx	3 (0.8)
M0	382 (99.2)
Stage grouping (TNM 7&8)	
1	38 (9.8)
2	124 (32.2)
3	221 (57.4)
x	2 (0.5)
Radiation therapy	
Temporarily interrupted∗	12 (3.1)
Prematurely stopped†	8 (2.1)
Delivered as planned	365 (94.8)
Chemotherapy interrupted or stopped	
Yes	41 (10.9‡)
No	335 (89.1‡)

∗ Radiation therapy was subsequently continued to full dose.

† Total dose not delivered.

‡ Percentage of total number of patients who had chemotherapy (n = 376).

All patients were treated with definitive RT, according to UK IMRT guidance. Table EA (available at https://doi.org/10.1016/j.ijrobp.2019.10.016) details the treatment delivered. Fifteen patients did not receive doublet chemotherapy owing to comorbidities, poor performance status, or patient choice. Treatment was temporarily interrupted in 12 patients (intercurrent illness [5], cytopenias [4], sepsis [1], emergency hernia repair [1], and compliance [1]) and stopped prematurely in 8 (small bowel obstruction [2], treatment toxicity [2], patient choice [2], unrelated intercurrent illness [1], and death from neutropenic sepsis [1]).

One patient died during treatment (of neutropenic sepsis), and 3 died within 6 months of completion of treatment (complications of chronic renal disease, pneumocystis with unknown HIV status, and pneumonia on a background of chronic obstructive pulmonary disease).

The median follow-up interval for survivors was 24.0 months (range, 2.0-59.7).

### Treatment outcomes

**Fig. 1:**
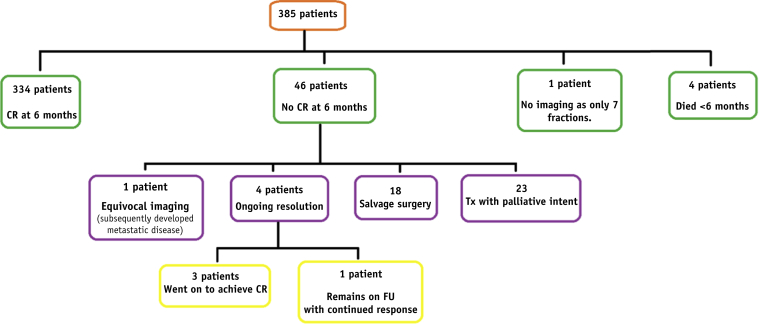
Flow chart of outcomes at 6 months post-completion of chemoradiation therapy.

Figure 1 illustrates the outcomes at 6 months post-CRT. Three hundred thirty-four patients (86.7%) achieved CR within 6 months of completing CRT. Disease recurrence at any point during follow-up occurred in 74 patients (19.2%). This included all persistent disease at the site of the primary tumor, within the pelvis or inguinal nodes, or subsequent development of new local, regional, or distant disease after a CR. The 3-year LRR was 19.5%, and 3-year distant relapse rate was 10.9%. The location of the first site of relapse is shown in Table 2. A summary of all patients with persistent or recurrent disease is available in supplementary materials, Table EB. (available online at https://doi.org/10.1016/j.ijrobp.2019.10.016).

**Table 2:** Patterns of treatment failure (93 sites of primary failure in 74 patients)

Site	n (% of total failures)
Locoregional∗ (± distant)	63 (85.1)
Primary site	62 (83.4)
Pelvic nodes	7 (9.5)
Inguinal nodes	5 (6.8)
Perineum	1 (1.4)
Distant† (± locoregional)	30 (40.5)
Lung	17 (23.0)
Liver	13 (17.6)
Distant nodes	13 (17.6)
Common iliacs	3 (4.1)
Paraortic	3 (4.1)
Bone	2 (2.7)
Adrenal	1 (1.4)
Subcutaneous tissues	1 (1.4)
Multiple distant organs	15 (20.3)

∗ Some patients experienced failures at multiple sites.

† First site of distant failure.

### Persistent disease

The 28 patients with residual tumor who did not receive salvage surgery did not receive surgery for the following reasons: inoperable disease (9), disseminated metastatic disease (7), unfit (4), or declined (2), imaging was thought to be equivocal because of a significant ongoing pressure sore (1), evidence of ongoing resolution of disease (4), death before treatment of residual disease could be considered (1). Of the responses stating inoperable, some were inoperable due to patient factors (eg, 1 patient was unwell with peritumoral sepsis, associated osteomyelitis, and hydronephrosis due to ureteric obstruction); most were surgical factors such as disease outside operative field, either primary extension or nodal deposits; some were factors demonstrating bad biology such as new satellite nodules in the right perineum or persistent inguinal disease.

### Sites of LRR after CR

Ten patients had isolated local recurrence at first site of relapse at a median of 17.7 months (range, 10.2-25.8) from the start of their CRT.

The 3-year incidence for regional recurrence was 14.1% (95% confidence interval [CI], 8.2-20.0) in patients with initially uninvolved nodes and 24.7% (95% CI, 18.2-31.2) in those with involved nodes at outset.

**Fig. 2:**
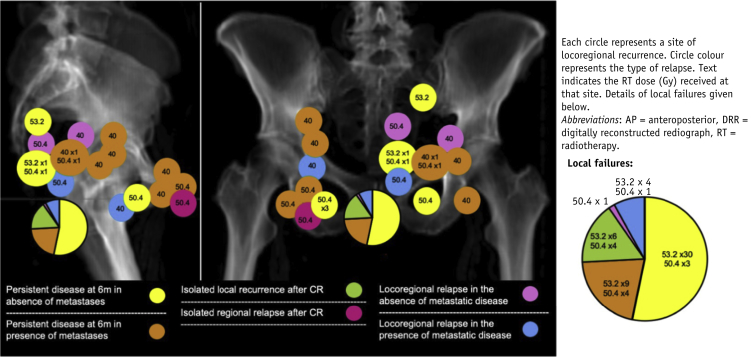
Representative plain kV films with locations of failure superimposed. All locoregional disease excluding persistent disease at the primary site is depicted.

Five patients had regional relapse in nodes that were not involved at outset in the presence of metastatic disease. One occurred in a patient with CR at 6 months, and the other 4 occurred in patients who failed to achieve CR at 6 months. Only 2 patients had regional relapse as first site of relapse in the absence of concurrent distant relapse. One patient relapsed in a previously involved left mesorectal lymph node and the second in a previously involved right inguinal node. Figure 2 demonstrates the locations of the locoregional failures.

### Distant disease

Overall, 30 patients developed distant disease (7.8%). The median time to development of distant disease was 14.6 months (range, 8.3-32.7) from the start of CRT. Distant metastases occurred in 13 of the 46 (28.3%) patients who failed to achieve a CR 6 months after CRT, compared with 17 of the 334 (5.1%) patients with a CR at 6 months.

### Salvage surgery

Eighteen of the 46 patients with documented incomplete response (39.1%) underwent salvage APR. Twelve of the 18 remained cancer free for the remainder of follow-up.

Of the 10 patients who had an isolated local recurrence after a documented CR to CRT, 8 underwent salvage APR. Seven achieved an R0 resection and remained cancer free for the remainder of follow-up. Two patients were treated with palliative intent due to patient choice and poor tumor biology.

One of the 2 patients with regional relapse in the absence of distant disease underwent salvage APR; however, this patient died of metastatic disease 11 months postsurgery.

### Survival

**Fig. 3:**
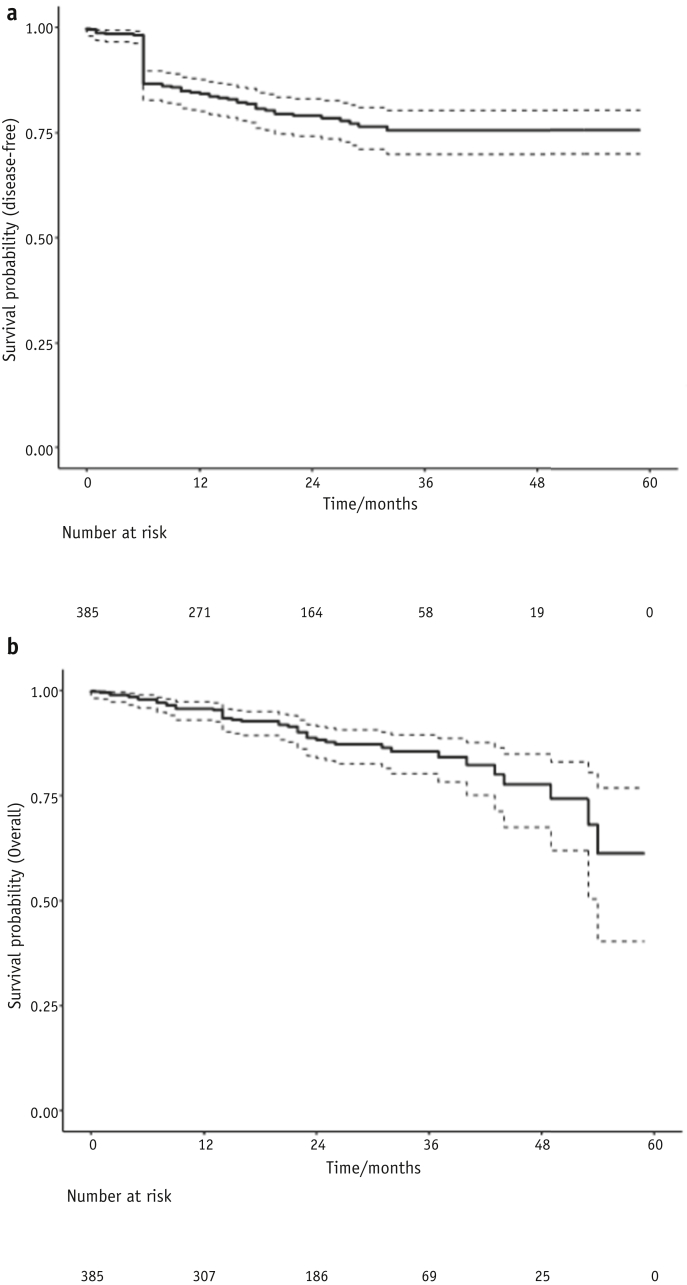
Disease-free (a) and overall (b) survival with confidence intervals.

There were a total of 47 deaths from any cause, including 36 (9.4%) from ASCC. Figure 3 shows Kaplan-Meier curves for disease-free and overall survival. Three-year overall survival was 85.6% (95% CI, 81.1-90.1), and 3-year disease-free survival was 75.6% (95% CI, 70.5-80.7). Median disease-free and median overall survival time were not reached due to relatively short follow-up.

The 3-year colostomy-free survival was 69.0% (95% CI, 63.1-74.1%).

### Predictors of outcome

Predictive factors for locoregional relapse included male sex, high N-stage, and failure to complete RT as planned (Table 3). To take the competing risk of death into account, we calculated the cumulative incidence of LRR.^27^ There was a negligible difference in the estimates between the methods: Using the Kaplan-Meier method, the incidences at 12, 24, and 36 months, respectively, were 15.1%, 18.6%, and 19.7% compared with 15.0%, 18.5%, and 19.6% using the cumulative incidence method.

**Table 3:** Predictors of locoregional recurrence (n = 385)

Variable	Value	Univariable		Multivariable	
		Hazard ratio (CI)	*P*	Hazard ratio (CI)	*P*
Age	Per year	1.01 (0.99-1.04)	.252	1.02 (0.99-1.04)	.187
Sex	Female	Reference		Reference	
	Male	1.78 (1.09-2.91)	.021	2.08 (1.24-3.48)	.005
Performance status	0	Reference		Reference	
	1	1.90 (1.14-3.19)	.014	1.31 (0.75-2.28)	.341
	2	2.09 (0.73-5.98)	.169	1.27 (0.40-4.00)	.680
	3	3.53 (0.84-14.93)	.086	2.78 (0.60-12.90)	.192
T stage	1	Reference		Reference	
	2	1.67 (0.50-5.66)	.407	1.26 (0.37-4.36)	.712
	3	4.13 (1.23-13.79)	.021	2.54 (0.72-8.95)	.148
	4	5.17 (1.55-17.28)	.008	2.87 (0.81-10.17)	.102
	x	0 (0-∞)	.996	0 (0-∞)	.998
N stage (TNM 7)	0	Reference		Reference	
	1	2.24 (1.19-4.26)	.013	2.23 (1.13-4.39)	.021
	2	1.37 (0.64-2.90)	.416	0.79 (0.34-1.82)	.576
	3	3.05 (1.63-5.73)	<.001	1.85 (0.91-3.75)	.088
	x	0 (0-∞)	.996	0 (0-∞)	.998
N stage (TNM 8)	0	Reference			
	1a	2.17 (1.28-3.71)	.004		
	1b	0 (0-∞)	.997		
	1c	2.19 (0.93-5.14)	.073		
	x	0 (0-∞)	.996		
RT completion	Completed as planned	Reference		Reference	
	Incomplete or interrupted∗	5.29 (2.83-9.90)	<.001	4.96 (2.40-10.27)	<.001
Chemotherapy	MMC 5FU	Reference		Reference	
	MMC capecitabine	0.92 (0.56-1.51)	.747	0.95 (0.56-1.58)	.830
	Single agent	1.24 (0.17-9.03)	.833	1.91 (0.25-14.62)	.532
	None	0.71 (0.10-5.15)	.732	0.57 (0.07-4.57)	.597

*Abbreviations:* 5FU = 5-fluorouracil; CI = 95% confidence interval; MMC = mitomycin-c; RT = radiation therapy.

∗ An interruption in radiation therapy was defined as any extension more than 2 days over the planned overall treatment time.

Three-year locoregional relapse for the 2 main chemotherapy regimens used was 19.0% (95% CI, 12.3-25.7) for mitomycin/capecitabine and 19.7% (95% CI, 15.6-23.8) for mitomycin/5-fluorouracil (5FU). Three-year distant relapse was 11.0% (95% CI, 4.7-17.3) and 7.1% (95% CI, 3.0-11.2) for mitomycin/capecitabine versus mitomycin/5FU, respectively. Lastly, the 3-year DFS between the 2 regimens was 81.0% (95% CI, 73.9-88.1) and 81.9% (95% CI, 76.0-87.8) in favor of mitomycin/5FU.

Predictors for other outcomes (distant relapse, persistent disease, disease-free and overall survival) were similar and are available in the supplementary materials (Tables EC to EF available online at https://doi.org/10.1016/j.ijrobp.2019.10.016).

## Discussion

To the best of our knowledge, this is the only series reporting patterns of failure after IMRT and the largest published series of IMRT examining outcomes. It should serve as a valuable resource for the future optimization of IMRT and the development and design of future trials incorporating IMRT.

The disease outcomes from IMRT outlined here are equivalent to those published from 3D conformal RT. Our reported CR rate (86.7%) is similar to the 90% seen in the ACT2 trial despite the inherently different populations treated in clinical trials.^11^ Our 3-year DFS (75.6%) is similar to the 73% reported in ACT2, and our overall survival (85.6%) is comparable with that in RTOG 9811, ACCORD, and ACT2 at 78%, 74%, and 85%, respectively.^9^, ^10^, ^11^

Comparison to other smaller IMRT series is difficult owing to the varying outcome definitions; however, 2-year, 3-year, and 4-year DFS is reported as 84.4%, 71%, and 82% in smaller published series.^28^, ^29^, ^30^, ^31^ In terms of patterns of relapse we can compare to the 4 retrospective cohorts of a 3D conformal technique, acknowledging that these publications reported different outcomes and definitions of outcomes.^32^, ^33^, ^34^, ^35^ Our LRR (19.5%) is similar to the 14.4% to 25% presented in these papers. In terms of percentage of all recurrences, our primary tumor recurrence rate (83.4%) was higher than in previous publications (40.9%-53.8%); however, both our pelvic relapse (9.5%) and inguinal relapse rate (6.8%) are at the lower end of what was previously reported. Possible hypotheses for this low pelvic relapse are as follows: 2 of the previous studies did not routinely include the inguinal nodes in the prophylactic volume, whereas our guidance advocates that all high-risk T1 and ≥T2 disease should undergo prophylactic inguinal irradiation; the increased homogeneity of coverage of at risk lymph nodes with IMRT; and finally, the increased routine use of 18-fluorodeoxyglucose PET results in more involved nodes than previously. Tumor control probability modeling data suggest higher doses result in improved tumor control^36^; therefore, if we are finding more involved nodes and delivering a higher boost dose to them, this may lower regional relapse rates.

Our rate of distant relapses (7.8%) is lower than those previously reported. This may be due to the improved staging at outset with PET or short follow-up. The increased distant metastasis rate in those with an incomplete response after CRT may suggest this group with radioresistant disease is a different biological subgroup and continued interrogation of tumor biology is required.^37^

Consistent with Bentzen et al,^35^ a substantial proportion of those with residual tumor were not candidates for salvage surgery. Previous series have shown that a significant proportion of patients who have salvage surgery subsequently relapse and ultimately die of ASCC.^35^ Our data show that 73.1% who underwent salvage surgery remain alive and free from disease. Accepting limited follow-up and more sophisticated assessment of occult metastatic disease (with PET), the high R0 resection rate indicates good preoperative assessment and surgical approach.

Some insight can be gained into the dose used for prophylactic nodes. We delivered 40 Gy in 28 fractions, which is a biologically equivalent dose equivalent to 30.6 Gy in 17 fractions delivered in the ACT2 trial. For calculations we used an alpha-beta ratio of 8 and a loss of 0.7 Gy per day after 20 fractions.^38^ With this dose, there were only 2 relapses in regional nodes in the absence of metastatic disease. Both occurred in nodes that were present and boosted at outset. Internationally there has been a move to deliver much higher prophylactic doses to the noninvolved nodes. Our series suggests that 40 Gy in 28 fractions is sufficient to prevent regional disease in prophylactic nodal groups.

In terms of informing IMRT delineation, an area of controversy is whether to include the IRF in the prophylactic volume. UK guidelines only include the IRF when disease is there at outset. This would appear sufficient considering there was 1 perineal relapse, in a patient who had T4 disease at outset, which may have included the IRF. The superior border is another area of uncertainty and differs between protocols. With our superior border, we had no relapses in internal iliac/external iliac nodes above our border, in the absence of widespread metastatic disease.

A further observation was made regarding the new TNM 8 classification. In our series only 1 case fulfilled the N1b criteria. Although the differentiation between N0 and N1a or N1c was very predictive, the number of patients within N1b suggests this criterion may be of limited use or difficult to validate.

The results of the predictive factors are in keeping with literature to date other than the lack of T stage affecting DFS or local relapse on multivariant analysis.^32^^,^^33^ The authors found it hard to identify a hypothesis for this finding. It may be that T stage in historic papers was associated with regional nodal relapse and because this occurred less in this series it is no longer predictive. Further work would be required to identify whether this is a true finding. The correlation of N stage with distant recurrence may warrant further investigation into newer chemotherapeutic, immunotherapeutic, and targeted agents in patients with node-positive ASCC, such as the CORINTH study, a phase 1 trial investigating the use of pembrolizumab concurrently with CRT: (EudraCT 2017-002300-27) and the adjuvant nimvolumab study (NCT03233711).

The almost identical LRR and DFS for the 2 different main chemotherapy regimens used is a similar finding to that published using the data from the UK anal cancer audit.^39^ The significantly poorer outcomes experienced by patients who failed to complete RT as planned emphasizes the importance of close monitoring and management of toxicities during treatment.

Our study has limitations. It was performed retrospectively, based on data collected from hospital records. Despite this we have collected all data, excluding 1.8% of performance status. A number of patients (38.7%) were not tested for HIV because this was not routine in all centers at the time of treatment. Unfortunately, human papillomavirus testing was not performed in any of the centers over this period, so we cannot comment on this. As mentioned in the Methods and Materials section, the different centers had different thresholds of use for PET. The center that performed PET scans on all ≥T2 patients contributed 28.3% of the patients, and the center that had no access contributed 18.4% of all patients. The other 3 centers with selective use of PET scanning contributed 53.2% in total. Hence, although PET was not standard it was used in a larger cohort of patients than in previous studies, likely affecting results as mentioned throughout the Discussion. Our series did not involve quality assurance of RT planning or delivery, so adherence to the guidance cannot be guaranteed. However all 5 centers involved were involved in a number of meetings to design the guidance. Once published, there were further meetings to advertise and disseminate the guidance and finally contouring workshops over this period in the run up to the PLATO study. At all of these points, contours were reviewed and discussed. Four of the centers were involved in delineating the gold standard for use in Radiotherapy Trials Quality Assurance in PLATO, and 3 were centers that provide contour review for PLATO study. Different strategies for identification and management of toxicities in different centers could have affected completion rates and subsequently outcomes. Unfortunately, due to the different populations in the different centers, the authors believed an analysis between centers would have too many confounding factors. There is the possibility of some patient selection at the level of each center.

It must be acknowledged that these patients were treated with the IMRT delivery according to UK guidance and as such results are not applicable to other doses, volumes, and constraints.

Some caution is required in interpreting the outcomes of treatment due to the relatively short follow-up, 2 years compared with 4 for other series.^32^, ^33^, ^34^, ^35^ A number of our patients (45.5%) have <2 years follow-up. Long-term outcome evaluations have previously shown that 84% of failures after CRT for ASCC occur in the first 2 years^40^ and 94% by 3 years.^41^ Whether the pattern of late relapse is different from the pattern of early relapse has not previously been studied and similarly cannot be answered by this series. However, late relapses are rare, so the authors believe these data offer a useful focus for further research and development.

In terms of statistical analysis, although for this rare tumor type we have achieved a large data set, for statistical purposes it is relatively small based on the number of events for each outcome. These predictive models should therefore be interpreted with caution because they are underpowered for the intended number of variables for testing.

The toxicity of treatment is not addressed by our series. The acute toxicity of our guidance has been previously published in a prospective series.^22^ Late toxicity of treatment is a factor we did not investigate owing to the limitations of retrospective reviews of late toxicity. Work is ongoing with the UK anal cancer audit to analyze prospectively gathered late toxicity using Patient Reported Outcome Measures, which we hope will contribute significantly to this important question.

Lastly, there are significant difficulties in comparison across trials stemming from the varying outcome definitions. We defined our clinical outcomes with advice from the CORMAC team, who recently published a core set of outcomes in ASCC^42^ with the inclusion of the additional outcome colostomy-free survival due to international interest in this outcome. The CORMAC group undertook a systematic review of anal cancer trials, identifying 1192 different anal cancer outcomes. Subsequent to this they published international patient and health care professional consensus outcomes. Although the definitions of these outcomes are not agreed on, we have acknowledged the CORMAC group, who assisted in the definition of the outcomes reported. These are also in keeping with the ongoing PLATO trial.^43^

The IMRT guidance was developed in the run up to the PLATO trial, which is a platform of studies for ASCC using the UK IMRT guidance as a backbone for RT delivery. The patterns of recurrence reported here support the rationale of the ACT5 trial. It evaluates whether dose escalation to the sites of gross tumor reduces locoregional failure.

## Conclusions

This series provides much-needed data on the patterns of relapse after IMRT in ASCC. We believe this series supports the use of the UK IMRT guidance in routine clinical care. The lower prophylactic nodal dose of 40 Gy in 28 fractions is sufficient to prevent isolated regional relapse in uninvolved nodes. Due to the high rate of relapse at the primary site, strategies to optimize the radiation response, such as dose escalation, immune modulation, or radiosensitization, are most likely to have an impact on disease-free survival and overall survival.

## Supplementary Data

Tables EA-EJ
