# Supplementary material for: Patterns and Predictors of Relapse Following Radical Chemoradiation Therapy Delivered Using Intensity Modulated Radiation Therapy With a Simultaneous Integrated Boost in Anal Squamous Cell Carcinoma
Source: Int J Radiat Oncol Biol Phys. 2020 Feb 1;106(2):329–39. doi: 10.1016/j.ijrobp.2019.10.016 (PMC6961207; doi:10.1016/j.ijrobp.2019.10.016)
Supplement: Tables EA-EJ [file mmc1.docx]

**Supplementary Materials**

| **Table A** Treatment characteristics (n=385) | |
| --- | --- |
|  | n (% of total) |
| Pre-chemoradiation colostomy |  |
| Yes | 55 (14.3) |
| No | 330 (85.7) |
| Dose to primary disease |  |
| 53.2Gy | 258 (67.0) |
| 50.4Gy | 127 (33.0) |
| Dose to nodal disease |  |
| 53.2Gy | 5 |
| 50.4Gy | 194 |
| Dose to elective nodal regions |  |
| 40Gy | 372 (96.7) |
| 39.2Gy | 13 (3.4) |
| Ischiorectal fossa included |  |
| Yes | 103 (26.8) |
| No | 282 (73.2) |
| Chemotherapy interrupted or stopped | |
| Yes | 41 (10.9*) |
| No | 335 (89.1*) |
| MMC = mitomycin-C; 5FU = 5-Fluorouracil.  * percentage of total number of patients who had chemotherapy (n = 376) | |

| **Table B** Clinical outcomes for patients with persistent or recurrent disease (n=74) | | | | | | |
| --- | --- | --- | --- | --- | --- | --- |
| No. | Age at dx | T stage | N stage* | Site | Treatment** | Status |
| 1 | 79 | 3 | 2 | Anus | Surgery | Dead; non-cancer |
| 2 | 57 | 2 | 1 | Anus, mesorectum, pelvic sidewall, liver, abdominal wall | Surgery | Dead; metastatic |
| 3 | 76 | 2 | 0 | Anus | Surgery | Alive; disease-free |
| 4 | 72 | 3 | 3 | Lung | Chemotherapy | Dead; metastatic |
| 5 | 63 | 1 | 0 | Anus | Surgery | Alive; disease-free |
| 6 | 71 | 4 | 3 | Mediastinum, lung | Chemotherapy | Dead; metastatic |
| 7 | 65 | 3 | 0 | Common iliac | Chemotherapy | Dead; metastatic |
| 8 | 84 | 3 | 0 | Anus | Surgery | Alive; disease-free |
| 9 | 50 | 2 | 1 | Liver, retroperitoneal nodes | Chemotherapy | Dead; metastatic |
| 10 | 69 | 4 | 1 | Anus, liver | Chemotherapy | Alive; metastatic |
| 11 | 72 | 3 | 3 | Anus | Chemotherapy | Alive; local disease |
| 12 | 66 | 2 | 0 | Anus | Surgery | Alive; disease-free |
| 13 | 58 | 4 | 3 | Liver | Surgery | Alive; disease-free |
| 14 | 63 | 1 | 3 | Lung, mediastinum, retroperitoneal nodes | Chemotherapy | Dead; metastatic |
| 15 | 68 | 2 | 1 | Liver, bone | Chemotherapy | Alive; metastatic |
| 16 | 72 | 3 | 2 | Epigastric nodes | Best supportive care | Dead; metastatic |
| 17 | 62 | 4 | 1 | Anus, liver | Chemotherapy | Alive; metastatic |
| 18 | 80 | 3 | 1 | Anus | Surgery (R1) | Alive; local disease |
| 19 | 46 | 1 | 2 | R inguinal | Chemotherapy | Dead; metastatic |
| 20 | 62 | 2 | 0 | Anus | Surgery | Alive; disease-free |
| 21 | 80 | 4 | 3 | Lung, para-aortics, | Active monitoring | Dead; metastatic |
| 22 | 76 | 4 | 0 | Anus | Surgery | Alive; disease-free |
| 23 | 70 | 2 | 0 | Anus, liver | Chemotherapy | Alive; metastatic |
| 24 | 66 | 3 | 3 | Anus, liver, lung | Surgery | Dead; metastatic |
| 25 | 64 | 2 | 0 | Anus | Best supportive care | Dead; local disease |
| 26 | 49 | 2 | 3 | Lung | Surgery | Alive; metastatic |
| 27 | 45 | 2 | 1 | Anus, mesorectum, mediastinum, lung | Chemotherapy | Alive; metastatic |
| 28 | 54 | 2 | 2 | Liver | Non-surgical management | Alive; metastatic |
| 29 | 82 | 3 | 1 | Refractory primary | Nil: not fit for surgery | Alive; local disease |
| 30 | 45 | 3 | 3 | Refractory primary, pelvic nodes, liver, adrenal, lung | Non-surgical management | Alive; local disease |
| 31 | 70 | 2 | 3 | Refractory primary, common iliac, retroperitoneal, lung, neck nodes | Chemotherapy | Alive; metastatic |
| 32 | 64 | 4 | 2 | Refractory primary | Non-surgical management | Dead; local disease |
| 33 | 63 | 4 | 3 | Refractory primary, perineum | Nil: not fit for surgery | Alive; local disease |
| 34 | 64 | 3 | 2 | Refractory primary, mesorectum | Nil: patient declined | Alive; local disease |
| 35 | 44 | 4 | 3 | Refractory primary | Nil: inoperable | Alive; local disease |
| 36 | 67 | 4 | 1 | Refractory primary, liver | Non-surgical management | Alive; metastatic |
| 37 | 48 | 4 | 3 | Refractory primary, mesorectum, R inguinal | Nil: inoperable | Alive; local disease |
| 38 | 47 | 4 | 3 | Refractory primary | Nil: inoperable | Alive; local disease |
| 39 | 50 | 4 | 1 | Refractory primary, pelvic nodes | Chemotherapy | Dead; local disease |
| 40 | 81 | 4 | 2 | Refractory primary, lung, liver | Non-surgical management | Dead; metastatic |
| 41 | 58 | 2 | 1 | Refractory primary | Surgery | Alive; disease-free |
| 42 | 71 | 3 | 1 | Refractory primary | Surgery | Dead; local disease |
| 43 | 60 | 2 | 0 | Refractory primary, lung | Surgery, Chemotherapy | Dead; metastatic |
| 44 | 59 | 3 | 0 | Refractory primary, R inguinal, lung | Non-surgical management | Dead; metastatic |
| 45 | 68 | 4 | 1 | Refractory primary, retroperitoneal nodes, lung, SCF | Radiotherapy (to PAN) | Dead; metastatic |
| 46 | 52 | 3 | 3 | Refractory primary, PA nodes | Non-surgical management | Dead; metastatic |
| 47 | 49 | 3 | 3 | Refractory primary, R inguinal | Surgery | Dead; local disease |
| 48 | 68 | 3 | 2 | Refractory primary, bilateral pelvic, inguinal, common iliac and PA nodes | Non-surgical management | Dead; metastatic |
| 49 | 47 | 2 | 0 | Refractory primary, lung | Surgery | Alive; metastatic |
| 50 | 74 | 4 | 0 | Refractory primary | Nil: unfit for surgery | Dead; local disease |
| 51 | 74 | 4 | 1 | Refractory primary, lung | Non-surgical management | Dead; metastatic |
| 52 | 55 | 3 | 1 | Refractory primary, bilateral pelvic nodes, liver, hilar nodes, lung | Surveillance (local disease related to pressure sore?) | Dead; metastatic |
| 53 | 68 | 4 | 2 | Refractory primary | Nil: inoperable | Dead; local disease |
| 54 | 41 | 4 | 1 | Refractory primary | Nil: patient non-compliant with primary CRT | Alive; local disease |
| 55 | 69 | 4 | 2 | Refractory primary | Nil: unfit for surgery, non-compliant | Dead; local disease |
| 56 | 64 | 3 | 0 | Refractory primary | Surgery | Alive; disease-free |
| 57 | 56 | 3 | 2 | Refractory primary | Surgery | Alive; disease-free |
| 58 | 64 | 2 | 0 | Refractory primary | Surgery | Alive; disease-free |
| 59 | 68 | 3 | 3 | Delayed response | Surveillance | Alive; local disease |
| 60 | 74 | 3 | 2 | Refractory primary | Surgery | Alive; disease-free |
| 61 | 63 | 4 | 3 | Refractory primary, retroperitoneal and pericardiac nodes, lung, bone | Non-surgical management | Alive; metastatic |
| 62 | 56 | 4 | 3 | Refractory primary | Nil: inoperable | Alive; local disease |
| 63 | 85 | 2 | 2 | Delayed response | Surveillance | Alive; disease-free |
| 64 | 75 | 4 | 0 | Refractory primary | Nil: inoperable | Alive; local disease |
| 65 | 59 | 2 | 0 | Refractory primary | Nil: patient died prior to treatment initiation | Dead; local disease |
| 66 | 58 | 3 | 0 | Refractory primary | Surgery (R1) | Alive; local disease |
| 67 | 59 | 2 | 3 | Refractory primary | Surgery | Alive; disease-free |
| 68 | 78 | 4 | 1 | Refractory primary | Surgery | Alive; disease-free |
| 69 | 54 | 2 | 0 | Refractory primary | Surgery | Dead; non-cancer |
| 70 | 56 | 1 | 0 | Refractory primary | Surgery | Alive; disease-free |
| 71 | 54 | 2 | 1 | Refractory primary | Surgery | Dead; non-cancer |
| 72 | 73 | 2 | 0 | Refractory primary | Surgery | Alive; disease-free |
| 73 | 61 | 3 | 3 | Refractory primary | Surgery | Alive; disease-free |
| 74 | 77 | 4 | 3 | Delayed response | Surveillance | Alive; disease-free |
| *TNM 7 | | | | | | |
| **Initial treatment choice | | | | | | |

| **Table C** Predictors of distant recurrence. (n = 385)  RT = radiotherapy; CI = confidence interval. | | | | | |
| --- | --- | --- | --- | --- | --- |
|  |  | *Univariable* | | *Multivariable* | |
| Variable | Value | Hazard ratio (CI) | *p* | Hazard ratio (CI) | *p* |
| Age | Per year | 1.01 (0.98-1.04) | 0.610 | 1.02 (0.99-1.06) | 0.191 |
| Sex | Female | Reference |  | Reference |  |
|  | Male | 2.10 (0.98-4.49) | 0.055 | 2.41 (1.09-5.35) | 0.030 |
| Performance status | 0 | Reference |  | Reference |  |
|  | 1 | 1.55 (0.73-3.29) | 0.259 | 1.13 (0.50-2.58) | 0.766 |
|  | 2 | 0.00 (0.00-Inf) | 0.997 | 0 (0-Inf) | 0.998 |
|  | 3 | 0.00 (0.00-Inf) | 0.999 | 0 (0-Inf) | 0.999 |
| T stage | 1 | Reference |  | Reference |  |
|  | 2 | 2.63 (0.34-20.56) | 0.357 | 1.92 (0.24-15.73) | 0.542 |
|  | 3 | 4.01 (0.49-32.60) | 0.194 | 1.74 (0.20-14.92) | 0.615 |
|  | 4 | 5.93 (0.75-46.84) | 0.091 | 2.59 (0.30-22.02) | 0.384 |
|  | x | 0 (0-$\infty$) | 0.998 | 0 (0-$\infty$) | 1.000 |
| N stage  (TNM 7)^$^ | 0 | Reference |  | Reference |  |
|  | 1 | 5.72 (1.72-19.00) | 0.004 | 5.45 (1.58-18.81) | 0.007 |
|  | 2 | 2.86 (0.71-11.42) | 0.138 | 2.36 (0.57-9.84) | 0.239 |
|  | 3 | 9.87 (3.14-31.01) | <0.001 | 8.50 (2.52-28.72) | <0.001 |
|  | x | 0 (0-$\infty$) | 0.998 | 0 (0-$\infty$) | 1.000 |
| N stage  (TNM 8)^$^ | 0 | Reference |  |  |  |
|  | 1a | 5.51 (1.87-16.31) | 0.002 |  |  |
|  | 1b | 182.80 (18.56-1800.86) | <0.001 |  |  |
|  | 1c | 6.26 (1.56-25.03) | 0.010 |  |  |
|  | x | 0 (0-$\infty$) | 0.998 |  |  |
| RT completion | Completed as planned | Reference |  | Reference |  |
|  | Incomplete or interrupted* | 3.05 (0.92-10.14) | 0.069 | 3.30 (0.88-12.44) | 0.078 |
| Chemotherapy | MMC 5-FU | Reference |  | Reference |  |
|  | MMC capecitabine | 1.64 (0.76-3.55) | 0.209 | 1.56 (0.71-3.44) | 0.272 |
|  | Single agent | 0.00 (0.00-Inf) | 0.998 | 0.00 (0.00-Inf) | 1.000 |
|  | None | 0.00 (0.00-Inf) | 0.997 | 0.00 (0.00-Inf) | 1.000 |
| *Abbreviation:* CI = 95% confidence interval | | | |  |  |
| *An interruption in radiotherapy was defined as any extension more than 2 days over the planned overall treatment time  ^$^ TNM stage was used in multivariant analysis for distant relapse as the small number of relapses that almost all occurred in AJCC Stage III group make the analysis uninterpretable if AJCC Stage is used. | | | | | |

| **Table D** Predictors of persistent disease at 6 months (n = 385)  RT = radiotherapy; CI = confidence interval | | | |  |  |
| --- | --- | --- | --- | --- | --- |
|  |  | *Univariable* | | *Multivariable* | |
| Variable | Value | Odds ratio (CI) | *p* | Odds ratio (CI) | *p* |
| Age | Per year | 1.00 (0.98-1.03) | 0.845 | 1.00 (0.97-1.03) | 0.847 |
| Gender | Female | Reference |  | Reference |  |
|  | Male | 2.35 (1.28-4.32) | 0.006 | 2.62 (1.29-5.36) | 0.008 |
| Performance status | 0 | Reference |  | Reference |  |
|  | 1 | 2.90 (1.52-5.72) | 0.002 | 1.88 (0.90-3.99) | 0.094 |
|  | 2 | 3.27 (0.86-10.32) | 0.056 | 1.75 (0.34-7.31) | 0.469 |
|  | 3 | 6.13 (0.81-34.00) | 0.045 | 5.13 (0.50-43.78) | 0.137 |
| T stage | 1 | Reference |  | Reference |  |
|  | 2 | 3.33 (0.63-61.53) | 0.254 | 2.42 (0.42-45.93) | 0.413 |
|  | 3 | 10.95 (2.15-200.27) | 0.022 | 5.57 (0.96-105.88) | 0.114 |
|  | 4 | 16.1 (3.15-295.51) | 0.008 | 8.15 (1.40-155.63) | 0.054 |
|  | x | 0.00 (NA-2.11E+61) | 0.990 | 0 (NA-2.29E+103) | 0.993 |
| N stage (TNM 7) | 0 | Reference |  | Reference |  |
|  | 1 | 2.65 (1.13-6.15) | 0.023 | 2.15 (0.84-5.50) | 0.107 |
|  | 2 | 2.09 (0.82-5.10) | 0.109 | 1.54 (0.54-4.23) | 0.403 |
|  | 3 | 4.70 (2.12-10.70) | <0.001 | 2.43 (0.94-6.29) | 0.066 |
|  | x | 0 (NA-8.03E+72) | 0.993 | 0 (NA-4.49E+122) | 0.992 |
| N stage (TNM 8) | 0 | Reference |  |  |  |
|  | 1a | 2.99 (1.54-6.13) | 0.002 |  |  |
|  | 1b | 0 (NA-1.38E+123) | 0.993 |  |  |
|  | 1c | 3.31 (1.08-9.25) | 0.027 |  |  |
|  | x | 0 (NA-1.38E+123) | 0.993 |  |  |
| RT completion | Completed as planned | Reference |  | Reference |  |
|  | Incomplete or interrupted* | 7.36 (2.90-18.56) | <0.0001 | 5.80 (1.96-17.29) | 0.001 |
| Chemotherapy | MMC 5-FU | Reference |  | Reference |  |
|  | MMC capecitabine | 1.11 (0.59-2.03) | 0.749 | 1.35 (0.67-2.71) | 0.400 |
|  | Single agent | 1.4 (0.07-9.12) | 0.762 | 2.39 (0.11-23.02) | 0.484 |
|  | None | 0.88(0.05-5.04) | 0.902 | 0.62 (0.03-5.24) | 0.703 |
| *Abbreviation:* CI = 95% confidence interval; RT = radiotherapy; MMC = mitomycin-c; 5-FU = 5-fluorouracil | | | | | |
| *An interruption in radiotherapy was defined as any extension more than 2 days over the planned overall treatment time | | | | | |

| **Table E** Predictors of disease-free survival (n = 385)  RT = radiotherapy; CI = confidence interval. | | | |  |  |
| --- | --- | --- | --- | --- | --- |
|  |  | *Univariable* | | *Multivariable* | |
| Variable | Value | Hazard ratio (CI) | *p* | Hazard ratio (CI) | *p* |
| Age | Per year | 1.01 (0.99-1.04) | 0.166 | 1.02 (1.00-1.04) | 0.064 |
| Gender | Female | Reference |  | Reference |  |
|  | Male | 1.85 (1.18-2.92) | 0.008 | 2.16 (1.34-3.48) | 0.002 |
| Performance status | 0 | Reference |  | Reference |  |
|  | 1 | 1.94 (1.21-3.12) | 0.006 | 1.30 (0.78-2.16) | 0.313 |
|  | 2 | 1.83 (0.65-5.17) | 0.257 | 1.19 (0.38-3.66) | 0.759 |
|  | 3 | 3.32 (0.79-13.92) | 0.101 | 2.83 (0.62-12.82) | 0.178 |
| T stage | 1 | Reference |  | Reference |  |
|  | 2 | 1.53 (0.53-4.41) | 0.435 | 1.18 (0.40-3.48) | 0.767 |
|  | 3 | 3.61 (1.26-10.37) | 0.017 | 2.05 (0.67-6.20) | 0.206 |
|  | 4 | 4.48 (1.56-12.87) | 0.005 | 2.38 (0.78-7.23) | 0.127 |
|  | x | 0 (0-$\infty$) | 0.996 | 0 (0-$\infty$) | 0.997 |
| N stage (TNM 7) | 0 | Reference |  | Reference |  |
|  | 1 | 2.47 (1.34-4.56) | 0.004 | 2.49 (1.30-4.78) | 0.006 |
|  | 2 | 1.58 (0.78-3.20) | 0.201 | 1.01 (0.47-2.18) | 0.979 |
|  | 3 | 4.02 (2.25-7.17) | <0.001 | 2.73 (1.43-5.21) | 0.002 |
|  | x | 0 (0-$\infty$) | 0.995 | 0 (0-$\infty$) | 0.997 |
| N stage (TNM 8) | 0 | Reference |  |  |  |
|  | 1a | 2.52 (1.51-4.19) | <0.001 |  |  |
|  | 1b | 12.09 (1.62-90.27) | 0.015 |  |  |
|  | 1c | 2.73 (1.26-5.93) | 0.011 |  |  |
|  | x | 0 (0-$\infty$) | 0.996 |  |  |
| RT completion | Completed as planned | Reference |  |  |  |
|  | Incomplete or interrupted* | 4.98 (2.74-9.05) | <0.001 | 4.50 (2.26-8.97) | <0.001 |
| Chemotherapy | MMC 5-FU | Reference |  | Reference |  |
|  | MMC capecitabine | 1.08 (0.69-1.70) | 0.740 | 1.09 (0.68-1.75) | 0.719 |
|  | Single agent | 1.17 (0.16-8.53) | 0.875 | 1.79 (0.24-13.57) | 0.575 |
|  | None | 0.66 (0.09-4.83) | 0.686 | 0.49 (0.06-3.84) | 0.497 |
| *Abbreviation:* CI = 95% confidence interval; RT = radiotherapy; MMC = mitomycin-c; 5-FU = 5-fluorouracil | | | | | |
| *An interruption in radiotherapy was defined as any extension more than 2 days over the planned overall treatment time | | | | | |

| **Table F** Predictors of overall survival (n = 385) | | | |  |  |
| --- | --- | --- | --- | --- | --- |
|  |  | *Univariable* | | *Multivariable* | |
| Variable | Value | Hazard ratio (CI) | *p* | Hazard ratio (CI) | *p* |
| Age | Per year | 1.02 (0.99-1.04) | 0.262 | 1.01 (0.99-1.04) | 0.304 |
| Gender | Female | Reference |  | Reference |  |
|  | Male | 2.93 (1.64-5.24) | <0.001 | 4.00 (2.11-7.56) | <0.001 |
| Performance status | 0 | Reference |  | Reference |  |
|  | 1 | 2.97 (1.53-5.76) | 0.001 | 1.73 (0.84-3.56) | 0.137 |
|  | 2 | 7.53 (2.66-21.32) | <0.001 | 4.83 (1.47-15.91) | 0.010 |
|  | 3 | 11.61 (2.56-52.75) | 0.002 | 10.71 (1.94-58.95) | 0.006 |
| T stage | 1 | Reference |  | Reference |  |
|  | 2 | 0.94 (0.26-3.43) | 0.927 | 0.69 (0.18-2.66) | 0.587 |
|  | 3 | 3.28 (0.95-11.30) | 0.059 | 2.15 (0.58-8.02) | 0.254 |
|  | 4 | 4.15 (1.21-14.25) | 0.024 | 2.08 (0.54-7.99) | 0.284 |
|  | x | 0 (0-$\infty$) | 0.997 | 0 (0-$\infty$) | 0.999 |
| N stage (TNM 7) | 0 | Reference |  | Reference |  |
|  | 1 | 1.93 (0.84-4.48) | 0.124 | 2.32 (0.94-5.72) | 0.067 |
|  | 2 | 2.19 (0.97-4.95) | 0.058 | 1.42 (0.57-3.54) | 0.451 |
|  | 3 | 3.40 (1.59-7.27) | 0.002 | 2.16 (0.92-5.09) | 0.077 |
|  | x | 0 (0-$\infty$) | 0.997 | 0 (0-$\infty$) | 0.998 |
| N stage (TNM 8) | 0 | Reference |  |  |  |
|  | 1a | 2.53 (1.33-4.83) | 0.005 |  |  |
|  | 1b | 0 (0-Inf) | 0.998 |  |  |
|  | 1c | 2.06 (0.67-6.29) | 0.205 |  |  |
|  | x | 0 (0-$\infty$) | 0.998 |  |  |
| RT completion | Completed as planned | Reference |  | Reference |  |
|  | Incomplete or interrupted* | 6.21 (2.98-12.95) | <0.001 | 4.22 (1.78-10.00) | 0.001 |
| Chemotherapy | MMC 5-FU | Reference |  | Reference |  |
|  | MMC capecitabine | 0.84 (0.46-1.53) | 0.572 | 0.76 (0.40-1.46) | 0.409 |
|  | Single agent | 0 (0-Inf) | 0.996 | 0 (0-Inf) | 0.996 |
|  | None | 2.53 (0.59-10.73) | 0.209 | 0.78 (0.14-4.31) | 0.779 |
| *Abbreviation:* CI = 95% confidence interval; RT = radiotherapy; MMC = mitomycin-c; 5-FU = 5-fluorouracil | | | | | |
| *An interruption in radiotherapy was defined as any extension more than 2 days over the planned overall treatment time | | | | | |

| **Table G**  Correlation with tumour stage and outcomes. | | | | | |
| --- | --- | --- | --- | --- | --- |
|  | Stage group |  |  |  |  |
|  | (TNM 7 & 8) |  |  |  |  |
| Locoregional recurrence | 1 | Reference |  |  |  |
|  | 2 | 2.52 (0.58-10.98) | 0.217 | 2.14 (0.49-9.36) | 0.312 |
|  | 3 | 4.59 (1.12-18.88) | 0.035 | 3.56 (0.86-14.79) | 0.08 |
|  | x | 0 (0-∞) | 0.996 | 0 (0-∞) | 0.996 |
|  |  |  |  |  |  |
| Persistent disease at 6 months | 1 | Reference |  |  |  |
|  | 2 | 3.25 (0.59-60.55) | 0.269 | 2.7 (0.47-51.31) | 0.361 |
|  | 3 | 7.93 (1.64-142.86) | 0.044 | 6.27 (1.23-114.86) | 0.079 |
|  | x | 0 (0-7.035E+13) | 0.991 | 0 (0-1.4282E+14) | 0.991 |
|  |  |  |  |  |  |
| Disease-Free Survival | 1 | Reference |  |  |  |
|  | 2 | 2.69 (0.62-11.65) | 0.185 | 2.36 (0.54-10.25) | 0.253 |
|  | 3 | 5.55 (1.35-22.71) | 0.017 | 4.50 (1.09-18.55) | 0.037 |
|  | x | 0 (0-∞) | 0.996 | 0 (0-∞) | 0.995 |
|  |  |  |  |  |  |
| Overall survival | 1 | Reference |  |  |  |
|  | 2 | 3.40 (0.44-26.62) | 0.243 | 2.67 (0.34-21.17) | 0.353 |
|  | 3 | 7.56 (1.03-55.40) | 0.047 | 5.91 (0.79-44.08) | 0.083 |
|  | x | 0 (0-∞) | 0.997 | 0 (0-∞) | 0.998 |
|  |  |  |  |  |  |
|  |  |  |  |  |  |
|  |  |  |  |  |  |

| **Table H** Patient and tumour characteristics for  patients with persistent disease (n=46) | |
| --- | --- |
|  | n (% of total) |
| Sex |  |
| Female | 25 (54.3) |
| Male | 21 (45.7) |
| Age |  |
| Median | 63 years |
| Range | 41-85 years |
| HIV |  |
| Positive | 3 (6.5) |
| Negative | 26 (56.5) |
| Not tested | 17 (40.0) |
| Performance status |  |
| 0 | 15 (32.6) |
| 1 | 25 (54.3) |
| 2 | 3 (6.5) |
| 3 | 2 (4.3) |
| Not documented | 1 (2.2) |
| T stage |  |
| Tx | 0 (0) |
| T1 | 1 (2.2) |
| T2 | 11 (23.9) |
| T3 | 15 (32.6) |
| T4 | 19 (41.3) |
| N stage (TNM 7) |  |
| Nx | 0 (0) |
| N0 | 12 (26.1) |
| N1 | 11 (23.9) |
| N2 | 9 (20.0) |
| N3 | 14 (30.4) |
| N stage (TNM 8) |  |
| Nx | 0 (0) |
| N0 | 12 (26.1) |
| N1a | 29 (63.0) |
| N1b | 0 (0) |
| N1c | 5 (10.9) |
| M stage |  |
| Mx | 1 (2.2) |
| M0 | 45 (97.8) |

| **Table J** Treatment characteristics for patients with persistent disease (n=46) | |
| --- | --- |
|  | n (% of total) |
| Pre-chemoradiation colostomy |  |
| Yes | 15 (32.6) |
| No | 31 (67.4) |
| Dose to primary disease |  |
| 53.2Gy | 39 (84.8) |
| 50.4Gy | 7 (15.2) |
| Dose to nodal disease |  |
| 53.2Gy | 1 |
| 50.4Gy | 31 |
| Dose to elective nodal regions |  |
| 40Gy | 44 (95.7) |
| 39.2Gy | 2 (4.3) |
| Ischiorectal fossa included |  |
| Yes | 12 (26.1) |
| No | 34 (73.9) |
| Chemotherapy |  |
| MMC-5FU | 22 (47.8) |
| MMC-capecitabine | 22 (47.8) |
| 5FU alone | 1 (2.2) |
| MMC alone | 0 (0) |
| None | 1 (2.2) |
| Radiotherapy | |
| Temporarily interrupted* | 4 (8.7) |
| Prematurely stopped** | 3 (6.5) |
| Delivered as planned | 39 (84.8) |
| Chemotherapy interrupted or stopped | |
| Yes | 11 |
| No | 34 |
| * Radiotherapy was subsequently continued to full dose  ** Total dose not delivered  MMC = mitomycin-C; 5FU = 5-Fluorouracil. | |
